# Knowledge, Attitudes, and Practices of NICU Doctors and Nurses Toward Prevention and Control of Nosocomial Infection With Multidrug Resistant Organism

**DOI:** 10.3389/fped.2022.817030

**Published:** 2022-04-19

**Authors:** Jinyan Zhou, Shuohui Chen

**Affiliations:** Administration Department of Nosocomial Infection, The Children's Hospital, Zhejiang University School of Medicine, National Clinical Research Center for Child Health, Hangzhou, China

**Keywords:** neonatal intensive care unit (NICU), doctors and nurses, knowledge attitudes and practices (KAP), multidrug resistant organism (MDRO), nosocomial infection

## Abstract

**Background:**

Nosocomial infection with multidrug resistant organisms (MDRO) can directly influence the curative effect and the prognosis of neonates, bringing great difficulties to clinical treatment. As direct contacts of neonates, the knowledge, attitudes, and practices (KAP) of doctors and nurses are critical for the prevention and control of MDRO infection in neonatal intensive care units (NICUs).

**Purpose:**

This study describes the KAP of doctors and nurses in NICUs toward the prevention and control of nosocomial infection with MDRO and analyzes its influencing factors.

**Methods:**

This cross-sectional study used convenience sampling to survey 397 doctors and nurses from the NICU of 28 hospitals in 11 cities in Zhejiang Province, China. A univariate analysis and a multiple linear regression were used to analyze the data.

**Results:**

The median scoring rate (interquartile range) of the knowledge, attitude, and practice questionnaire was 0.86 (0.82–0.91), 0.98 (0.91–1.00), and 0.995 (0.97–1.00), respectively. However, the median scoring rate regarding “basic knowledge of MDRO” and “special prevention and control measures” was 0.50 in knowledge. The multiple linear regression showed that the influencing factors of KAP were hospital grade, professional title, gender, regular supervision, and training.

**Conclusions:**

There was still room for improvement in the KAP of NICU doctors and nurses, especially regarding the knowledge. Men, doctors and nurses in Grade II hospitals, and doctors and nurses with primary professional titles had worse KAP. Training and supervision helped improve the KAP.

**Relevance to Clinical Practice:**

To improve the KAP of doctors and nurses to enhance the prevention and control effect for the MDRO infection in NICUs, hospitals and departments should carry out targeted training and strengthen supervision, while Grade II hospitals, men, and doctors and nurses with primary professional titles need more attention.

## Introduction

Multidrug resistant organism (MDRO) mainly refers to bacteria that are resistant to 3 or more classes of antibiotics used at the same time in clinical treatment (1). Infants in the neonatal intensive care unit (NICU) are at high risk of MDRO nosocomial infection owing to their small gestational age, low body weight, poor resistance, critical condition, and high incidence of invasive procedures (2–4). A survey of 29 European countries found that the incidence of nosocomial infection in the NICU was 10.7%, which was much higher than that in the neonatal unit (3.5%) (5). Hospital data from developing countries indicated that resistance to the WHO-recommended regimen of gentamicin was common among pathogens responsible for neonatal infections, where 71% of Klebsiella isolates and 50% of *Escherichia coli* isolates were gentamicin-resistant (6). Bacterial infection is the significant reason for 2.9 million neonatal deaths annually (7, 8). In addition to death, a severe bacterial infection in the neonatal period also expose neonates to a high risk of disability (9). MDRO infection can directly influence the curative effect and the prognosis of neonates, bringing great difficulties to clinical treatment (10). Doctors and nurses are in regular direct contact with neonates. These contacts can directly contribute to the transmission and control of the MDRO infection (11, 12). Full mastery of the knowledge, attitudes, and practices (KAP) related to nosocomial infection is the basis for improving nosocomial infection prevention and control (13–15). However, few studies have focused on KAP toward the prevention and control of MDRO infection, specifically for NICU doctors and nurses. The present study aimed to describe the KAP of doctors and nurses in NICUs toward the prevention and control of MDRO infection and to identify factors that may influence KAP. The findings of this study may be used to provide a reference for future studies and to assist in the development of measures to improve the prevention and control behaviors of NICU doctors and nurses.

## Methods

The STROBE checklist for cross-sectional studies was used.

### Study Design and Participants

In this cross-sectional study, doctors and nurses of NICUs from hospitals of different grades in different cities in Zhejiang Province, China, were selected by convenience sampling to complete the questionnaires from 18 January 2021 to 24 January 2021. To be eligible, all participants had to be certified, doctors or nurses should be registered, and doctors or nurses should have at least 1 year of clinical experience in NICU. In studies exploring influencing factors, the sample size should be at least 5–10 times the number of variables, according to the requirements of the statistical variable analysis (16). In the present study, the number of independent variables was 30. Considering the participants lost to follow-up, the sample size was expanded by 20% of the original size. Therefore, the sample size in our study was 30^*^10^*^(1+20%)=360.

### Data Collection

“Wenjuan Xing” (https://www.wjx.cn/), which is a professional questionnaire survey platform, was used in this study through WeChat. Wechat is a popular and widely used social app in China. The head nurse of the NICUs distributed the questionnaires to doctors and nurses in the NICUs through WeChat *via* an electronic link. A total of 397 questionnaires were completed by doctors and nurses from NICUs of 28 hospitals in 11 cities in Zhejiang Province.

### Variables and Instruments

Three tools were used to investigate the basic characteristics of the study subjects, information about MDRO infection prevention and control in hospitals and departments, and the KAP of participants.

#### Basic Characteristics

Basic characteristics, including the city of residence, hospital grade, gender, age group, education level, job position, professional title, and appointment as clinical teacher or NICU nosocomial infection control personnel, were collected.

#### Information About MDRO Infection Prevention and Control in Hospitals and Departments

With reference to the “Technical Guidelines for the Prevention and Control of Nosocomial Infection with MDRO” (17) issued by the Ministry of Health of China, survey items were formulated to collect information about MDRO infection prevention and control in hospitals and departments under investigation. The items consist of two parts: MDRO nosocomial infection management status and MDRO monitoring status of hospitals and departments, including the management of MDRO nosocomial infection, the management of key links, personnel training, the monitoring of MDRO, and the testing capabilities of clinical microbiology laboratories.

The basic characteristics, as well as the information about MDRO infection prevention and control in hospitals and departments, were the possible influencing factors associated with KAP of NICU doctors and nurses toward the prevention and control of nosocomial infection with MDRO.

#### The KAP Questionnaire

The questionnaire was designed by referring to the “Technical Guidelines for the Prevention and Control of Nosocomial Infection with MDRO” (17) issued by the Ministry of Health of China, experts' consensus (18), and the World Health Organization (WHO) guidelines (19, 20), as well as the relevant study (21). The questionnaire consists of three parts that correspond to the knowledge, attitudes, and self-reported practices of prevention and control of nosocomial infection with MDRO. The content validity of the questionnaire was evaluated by seven nosocomial infection control experts (three nosocomial infection control doctors and four nosocomial infection control nurses). The item-level content validity index (I-CVI) coefficient for each item was >0.78, the scale-level content validity index/universal agreement (S-CVI/UA) coefficient was 0.96, and the scale-level content validity index/average (S-CVI/Ave) coefficient was 0.99, indicating good content validity (22). In a pilot trial of the questionnaire with 30 participants, the Cronbach's coefficient of the knowledge, attitude, and practice portions of the questionnaire was 0.847, 0.963, and 0.997, respectively. Based on the work characteristics of doctors and nurses and after soliciting expert opinions, the attitude questionnaires of doctors and nurses in this study were the same, while the knowledge and practice questionnaires of doctors and nurses were slightly different.

The knowledge questionnaire for doctors consisted of three domains, namely, basic knowledge of MDRO, hand hygiene, contact isolation, aseptic technique, cleaning and disinfection, outbreak control of nosocomial infection, special prevention and control measures, and rational application and management of antibacterial drugs, with 57 items in total. Nurses were instructed to answer only 44 of these items, which corresponded to those in the first six domains. For each item, answers were in the form of true or false. One point was awarded for a correct answer and zero point for a wrong answer. The total score of the knowledge questionnaire was 57 (44) points, and the higher the score, the higher the knowledge level of the doctors and nurses.

The attitude questionnaire consisted of three domains: the characteristics of MDRO infection, the importance of doctors and nurses in prevention and control of infection, and the attitude of doctors and nurses toward the prevention and control of infection. There were a total of 11 items, each of which had the following five options: “strongly disagree,” “disagree,” “neutral,” “agree,” or “strongly agree,” corresponding to 1–5 points, respectively. These options were ordered in the direction of a more positive attitude. The total score of the attitude questionnaire was 55 points, and the higher the score, the more positive the attitude of doctors and nurses.

The practice questionnaire for doctors consisted of 51 items and five domains that included hand hygiene, contact isolation, aseptic technique, cleaning and disinfection, and rational application and management of antibacterial drugs. The options for items were ordered by the degree of execution and included “never,” “sometimes,” “about half the time,” “most of the time,” and “always,” corresponding to 1–five points, respectively. There were 40 items in the practice questionnaire for nurses that did not include the last domain. The total score of the practice questionnaire was 255 (200) points, and the higher the score, the better the self-reported compliance of doctors and nurses in MDRO infection prevention and control behavior.

Since the total number of items in the knowledge and practice questionnaire was different between doctors and nurses, the scoring rate was used for the correlation analysis, the univariate analysis, and the multiple linear regression of KAP. The scoring rate was the actual score divided by the total score. The closer the scoring rate was to 1, the higher the KAP level of doctors and nurses.

### Data Analysis

SPSS 20.0 statistical software was used for data analysis. The categorical variables were expressed as percentages and were compared using the χ^2^ test or Fisher's exact test. The Kolmogorov–Smirnov test was used to determine whether the continuous variables were normally distributed. Normally distributed continuous variables were represented by mean ± standard deviation, while non-normally distributed continuous variables were represented by median (interquartile range, IQR). Continuous variables were compared by the Mann–Whitney U test or a one-way ANOVA according to their distribution. A bivariate correlation Pearson coefficient was used to test the correlation between normal distribution continuous variables, and a bivariate correlation Spearman coefficient was used to test the correlation between classification variables and non-normal distribution continuous variables. All the tests were two-sided and *p* ≤ 0.05 was considered statistically significant. The multiple linear regression was carried out for the significant variables from the univariate analysis to obtain the influencing factors of KAP.

### Ethical Approval

The study was approved by the Ethics Committee of the Children's Hospital, Zhejiang University School of Medicine (2021-IRB-025). At the beginning of the questionnaire, the purpose of the study and informed consent were introduced, informing the participants that the survey was conducted anonymously, and they were free to choose whether to fill in the questionnaire or not.

## Results

### Basic Characteristics

A total of 397 doctors and nurses participated in the survey, and their basic characteristics are shown in Table 1. In China, the qualifications of hospitals are evaluated based on the hospital scale, medical hardware equipment, talents and technical strength, scientific research direction, and so on. In our study, doctors and nurses from three levels of hospitals participated in the survey, namely Grade III class A, Grade III class B, and Grade II class A. Most of the participants were women, and the age of most participants was between 26 and 40 years. Most respondents had a bachelor's degree (81.4%). Among the participants, 31.8% were clinical teachers and 12.3% were nosocomial infection control personnel. In terms of job positions, nurses, head nurses, doctors, deputy chief of department, and chief of department were all involved, but most respondents were nurses, accounting for 78.8%. In terms of professional titles, nurse, nurse practitioner, nurse-in-charge, associate professor of nursing, professor of nursing, resident doctor, attending doctor, associate chief physician, and chief physician were all involved. Most of the involved nurses were nurse practitioners and nurses-in-charge, and most doctors were resident doctors and attending doctors. Primary titles included nurse, nurse practitioner, and resident doctor, with a total of 233 people, accounting for 58.7%; intermediate titles included nurse-in-charge and attending doctor, with a total of 131 people, accounting for 33.0%; senior titles included associate professor of nursing, professor of nursing, associate chief physician, and chief physician, with a total of 33 people, accounting for 8.3%.

**Table 1:** Basic characteristics of the study subjects (*n* = 397).

**Characteristic**		**n**	**%**
Hospital grade^a^	Grade III class A	153	38.5
	Grade III class B	125	31.5
	Grade II class A	119	30.0
Gender	Male	20	5.0
	Female	377	95.0
Age group, yr	18–25	74	18.6
	26–30	110	27.7
	31–40	172	43.3
	41–50	36	9.1
	51–60	5	1.3
Education level	Technical Secondary School	3	0.8
	Junior College	63	15.9
	Undergraduate	323	81.4
	Master	7	1.8
	PhD	1	0.3
Job position	nurses	313	78.8
	head nurses	27	6.8
	doctors	33	8.3
	deputy chief of department	3	0.8
	chief of department	8	2.0
	other positions	13	3.3
Professional title	nurse	76	19.1
	nurse practitioner	143	36.0
	nurse-in-charge	115	29.0
	associate professor of nursing	16	4.0
	professor of nursing	4	1.0
	resident doctor	14	3.5
	attending doctor	16	4.0
	associate chief physician	8	2.0
	chief physician	5	1.3
Employed as clinical teacher	Yes	126	31.7
	No	271	68.3
Employed as nosocomial infection control personnel	Yes	49	12.3
	No	348	87.7

a *In China, hospitals are identified as Grade III, Grade II, or Grade I, and each grade is further classified as class A, class B, or class C after evaluation. Grade III is superior to Grade II, and Grade II is superior to Grade I. Class A is superior to class B, and class B is superior to class C*.

### The KAP of NICU Doctors and Nurses

The KAP of doctors and nurses in NICUs toward the prevention and control of nosocomial infection with MDRO is shown in Table 2. As can be seen from the table, in terms of knowledge, doctors and nurses had a poor grasp of “basic knowledge of MDRO” and “special prevention and control measures” (the median scoring rate was 0.50), but had a good grasp of the other six domains (the median scoring rate was not <0.90 and the first quartile was not <0.80). In terms of attitude, the median average score of all three domains was 5, and in the first quartile, it was no <4, indicating that the attitude of the majority of doctors and nurses was “agree” or “strongly agree”. In terms of practice, the median mean score of all five domains was 5, and in the first quartile, it was no <4.7, indicating that the self-reported compliance of most doctors and nurses to prevention and control measures was “most of the time” or “always”.

**Table 2:** The knowledge, attitudes, and practices (KAP) of neonatal intensive care units (NICU)‡ doctors and nurses.

**KAP**	**Domin**	**Min**	**Max**	**Median**
				**(interquartile range)**
Knowledge (average scoring rate)	Basic knowledge	0.20	1.00	0.50 (0.50–0.90)
	Hand hygiene	0.60	1.00	1.00 (0.80–1.00)
	Contact isolation	0.43	1.00	0.93 (0.93–1.00)
	Aseptic technique	0.00	1.00	1.00 (1.00–1.00)
	Cleaning and disinfection	0.20	1.00	0.90 (0.80–0.90)
	Outbreak control of nosocomial infection	0.00	1.00	1.00 (1.00–1.00)
	Special prevention and control measures	0.00	1.00	0.50 (0.50–0.50)
	Rational application and management of antimicrobial drugs	0.82	1.00	0.91 (0.82–1.00)
Attitude (average score)	The characteristics of mdro infection	1.00	5.00	5.00 (4.50–5.00)
	The importance of doctors and nurses in prevention and control of infection	1.00	5.00	5.00 (5.00–5.00)
	The attitude of doctors and nurses toward prevention and control of infection	1.00	5.00	5.00 (4.00–5.00)
Practice (average score)	Hand hygiene	1.00	5.00	5.00 (4.79–5.00)
	Contact isolation	1.46	5.00	5.00 (4.85–5.00)
	Aseptic technique	3.00	5.00	5.00 (5.00–5.00)
	Cleaning and disinfection	3.00	5.00	5.00 (4.92–5.00)
	Rational application and management of antimicrobial drugs	3.00	5.00	5.00 (4.82–5.00)

*^†^Knowledge, Attitudes, and Practices*.

*^‡^Neonatal Intensive Care Unit*.

In general, the median scoring rate (IQR) of knowledge, attitude, and practice were 0.86 (0.82–0.91), 0.98 (0.91–1.00), and 0.995 (0.97–1.00), respectively. In the correlation analysis, attitude was positively correlated with practice (*r* = 0.27, *p* < 0.01), while knowledge was not significantly correlated with attitude (r = 0.07, *p* = 0.20) or practice (r = −0.04, *p* = 0.45).

### Influencing Factors Associated With the KAP

The univariate analysis results are shown in Table 3. The univariate analysis showed that knowledge was significantly correlated with gender, hospital grade, job position, professional title, hospital's emphasis on establishing a nosocomial infection management department, hospital's emphasis on the prevention and control of MDRO infection, and the department's emphasis on the prevention and control of MDRO infection (*p* < 0.05 or 0.01). Attitude was significantly correlated with gender, hospital's emphasis on establishing a nosocomial infection management department, hospital's emphasis on the prevention and control of MDRO infection, department's emphasis on the prevention and control of MDRO infection, regular supervision, the number of MDRO infection prevention and control training per year, and willingness to train and carry out decolonization treatment (p < 0.05). Self-reported practice was significantly correlated with gender, age group, professional title, whether employed as nosocomial infection control personnel, hospital's emphasis on establishing a nosocomial infection management department, hospital's emphasis on the prevention and control of MDRO infection, department's emphasis on the prevention and control of MDRO infection, rules and regulations, hospital's attention to the NICU, isolation ward, regular supervision, the number of MDRO infection prevention and control training per year, willingness to train and carry out active screening and decolonization treatment (*p* < 0.05).

**Table 3:** Univariate analysis of KAP.

		**Knowledge (scoring rate)**		**Attitude (scoring rate)**		**Practice (scoring rate)**	
**Factors**		**Median (interquartile range)/r**	***P***	**Median (interquartile range)/r**	***P***	**Median (interquartile range)/r**	***P***
Gender	Male	0.84(0.78–0.86)	**0.01**	0.91(0.80–1.00)	**0.04**	0.96(0.84–1.00)	**0.01**
	Female	0.86(0.82–0.92)		0.98(0.91–1.00)		0.996(0.97–1.00)	
Hospital grade	Grade III class A	0.86(0.82–0.93)	**0.04**	0.96(0.91–1.00)	0.94	0.995(0.97–1.00)	0.67
	Grade III class B	0.86(0.82–0.92)		0.98(0.89–1.00)		1.00(0.97–1.00)	
	Grade II class A	0.84(0.80–0.90)		0.98(0.89–1.00)		1.00(0.97–1.00)	
Age group, yr	18–25	0.86(0.80–0.91)	0.65	0.98(0.89–1.00)	0.38	1.00(0.97–1.00)	**0.01**
	26–30	0.85(0.82–0.91)		0.96(0.90–1.00)		1.00(0.98–1.00)	
	31–40	0.86(0.82–0.91)		0.98(0.91–1.00)		0.995(0.97–1.00)	
	41–50	0.85(0.82–0.91)		0.93(0.90–0.996)		0.98(0.95–1.00)	
	51–60	0.88(0.85–0.95)		0.96(0.85–0.99)		0.91(0.90–0.98)	
Education level	Technical Secondary School	0.89(0.84–0.91)	0.96	0.96(0.96–0.98)	0.72	0.995(0.97–0.998)	0.20
	Junior College	0.86(0.80–0.91)		0.98(0.85–1.00)		1.00(0.97–1.00)	
	Undergraduate	0.86(0.82–0.91)		0.98(0.91–1.00)		0.995(0.97–1.00)	
	Master	0.88(0.82–0.89)		0.95(0.85–1.00)		0.995(0.97–1.00)	
	PhD	0.86		0.84		0.83	
Job position	Doctor	0.84(0.79–0.88)	**0.02**	0.96(0.80–1.00)	0.20	0.996(0.93–1.00)	0.19
	Nurse	0.86(0.82–0.93)		0.98(0.91–1.00)		0.995(0.97–1.00)	
Professional title	Primary	0.84(0.80–0.91)	**0.03**	0.98(0.90–1.00)	0.25	1.00(0.98–1.00)	**<0.01**
	Intermediate	0.86(0.84–0.93)		0.98(0.91–1.00)		0.995(0.97–1.00)	
	Senior	0.86(0.82–0.91)		0.93(0.90–0.99)		0.97(0.91–0.996)	
Clinical teacher	Yes	0.86(0.82–0.93)	0.08	0.98(0.91–1.00)	0.58	0.995(0.97–1.00)	0.43
	No	0.86(0.82–0.91)		0.98(0.91–1.00)		1.00(0.97–1.00)	
NICU nosocomial infection control personnel	Yes	0.86(0.82–0.93)	0.28	0.96(0.93–1.00)	0.87	0.98(0.93–1.00)	**0.01**
	No	0.86(0.82–0.91)		0.98(0.89–1.00)		1.00(0.98–1.00)	
Independent nosocomial infection management department	Yes	0.86(0.82–0.91)	0.28	0.98(0.91–1.00)	0.997	0.995(0.97–1.00)	0.61
	No	0.82(0.81–0.86)		0.98(0.93–0.99)		1.00(0.91–1.00)	
Hospital's emphasis on the establishing of nosocomial infection management department	Averagely	0.83(0.82–0.84)	**0.01**	0.96(0.93–1.00)	**<0.01**	0.90(0.90–0.91)	**<0.01**
	Relatively	0.83(0.77–0.89)		0.90(0.80–0.98)		0.99(0.92–1.00)	
	Greatly	0.86(0.82–0.93)		0.98(0.93–1.00)		1.00(0.98–1.00)	
Hospital's emphasis on prevention and control of MDRO infections	Averagely	0.84(0.82–0.85)	**0.01**	0.93(0.78–0.96)	**<0.01**	0.91(0.90–0.95)	**<0.01**
	Relatively	0.84(0.77–0.89)		0.89(0.80–0.98)		0.99(0.92–1.00)	
	Greatly	0.86(0.82–0.93)		0.98(0.93–1.00)		1.00(0.98–1.00)	
Department's emphasis on the prevention and control of MDRO infections	Averagely	0.84(0.82–0.86)	**<0.01**	0.93(0.85–1.00)	**<0.01**	0.91(0.90–0.99)	**<0.01**
	Relatively	0.82(0.77–0.89)		0.85(0.80–0.97)		0.97(0.83–1.00)	
	Greatly	0.86(0.82–0.93)		0.98(0.93–1.00)		1.00(0.98–1.00)	
Software system	Yes	0.86(0.82–0.91)	0.64	0.98(0.91–1.00)	0.37	0.995(0.97–1.00)	0.39
	No	0.84(0.82–0.88)		1.00(0.93–1.00)		0.97(0.92–1.00)	
Rules and regulations	Yes	0.86(0.82–0.91)	0.98	0.98(0.91–1.00)	0.98	0.996(0.97–1.00)	**0.01**
	No	0.86(0.82–0.92)		0.96(0.88–1.00)		0.92(0.83–0.97)	
Hospital's attention to the NICU	Yes	0.86(0.82–0.91)	0.75	0.98(0.91–1.00)	0.56	0.996(0.97–1.00)	**<0.05**
	No	0.86(0.84–0.89)		1.00(0.87–1.00)		0.94(0.91–0.96)	
Isolation ward	Yes	0.86(0.82–0.91)	0.25	0.98(0.91–1.00)	0.65	0.996(0.97–1.00)	**<0.05**
	No	0.82(0.74–0.91)		0.97(0.80–1.00)		0.97(0.83–0.995)	
Regular supervision by hospital	Yes	0.86(0.82–0.91)	0.14	0.98(0.91–1.00)	0.21	0.995(0.97–1.00)	**<0.05**
	No	0.72		0.80		0.80	
Regular supervision by NICU	Yes	0.86(0.82–0.91)	0.40	0.98(0.91–1.00)	0.31	0.995(0.97–1.00)	0.23
	No	0.93		0.82		0.92	
The number of related training per year		−0.01	0.91	0.14	**0.01**	0.25	**<0.01**
Willingness to train	Yes	0.86(0.82–0.91)	0.70	0.98(0.91–1.00)	**<0.05**	0.996(0.97–1.00)	**0.03**
	No	0.85(0.81–0.89)		0.91(0.80–0.97)		0.97(0.89–0.99)	
Monitoring by hospital	Yes	0.86(0.82–0.91)	0.87	0.98(0.91–1.00)	0.38	0.995(0.97–1.00)	0.08
	No	0.88 (0.82–0.93)		0.93		0.90(0.83–0.97)	
Monitoring by NICU	Yes	0.86(0.82–0.91)	0.41	0.98(0.91–1.00)	0.35	0.995(0.97–1.00)	0.70
	No	0.83(0.76–0.91)		0.93(0.80–0.98)		0.99(0.84–1.00)	
Collecting and submitting specimens in time	Yes	0.86(0.82–0.91)	0.87	0.98(0.91–1.00)	0.38	0.995(0.97–1.00)	0.97
	No	0.88(0.82–0.93)		0.93		0.99(0.97–1.00)	
Carrying out active screening	Yes	0.86(0.82–0.91)	0.88	0.98(0.91–1.00)	0.76	1.00(0.97–1.00)	**<0.01**
	No	0.84(0.82–0.93)		0.96(0.93–1.00)		0.97(0.91–0.99)	
Carrying out decolonization treatment	Yes	0.86(0.82–0.93)	0.61	0.98(0.91–1.00)	**0.01**	1.00(0.98–1.00)	**<0.01**
	No	0.86(0.82–0.89)		0.95(0.84–1.00)		0.98(0.94–1.00)	
Timely feedback from the laboratory	Yes	0.86(0.82–0.91)	0.53	0.98(0.91–1.00)	0.92	0.995(0.97–1.00)	0.26
	No	0.82		0.96		0.93	
Timely announcement of laboratory reports	Yes	0.86(0.82–0.91)	0.80	0.98(0.91–1.00)	0.96	0.996(0.97–1.00)	0.08
	No	0.85(0.82–0.88)		0.96(0.91–1.00)		0.96(0.94–0997)	
Accurate and comprehensive announcement	Yes	0.86(0.82–0.91)	0.62	0.98(0.91–1.00)	0.71	0.995(0.97–1.00)	0.27
	No	0.87(0.82–0.89)		0.95(0.91–1.00)		0.97(0.94–1.00)	
Frequency of the announcement	≤ Semiannually	0.86(0.82–0.91)	0.40	0.98(0.91–1.00)	0.59	0.995(0.97–1.00)	0.84
	>Semiannually	0.88(0.84–0.93)		0.95(0.90–1.00)		0.995(0.94–1.00)	

*^†^Knowledge, Attitudes, and Practices. The bold values indicate all pvalues p ≤ 0.05 which are statistically significant*.

Collinearity diagnosis found that there was multicollinearity among hospital's emphasis on establishing a nosocomial infection management department, hospital's emphasis on the prevention and control of MDRO infection, department's emphasis on the prevention and control of MDRO infection (*r* > 0.70, *p* < 0.01), and between age groups and professional titles (*r* > 0.70, *p* < 0.01). After removing corresponding variables according to the collinear diagnosis results, the significant factors obtained from the univariate analyses were included in the multiple regression model (Table 4). The results showed that the influencing factors of knowledge were as follows: Grade III class B hospital (compared with Grade II class A hospital) (B = 0.02, *p* = 0.01), intermediate title (B = 0.02, *p* = 0.02), and senior title (B = 0.04, *p* = 0.01) (compared with primary title). No factors significantly affected attitude. The influencing factors of self-reported practice were as follows: being female (B = 0.05, *p* < 0.01) (compared with male), regular supervision (B = 0.14, *p* = 0.03), and the number of MDRO infection prevention and control training per year (B = 0.004, *p* = 0.05).

**Table 4:** Multiple regression of the KAP.

	**Factors**		**B**	**SE^**‡**^**	**Beta**	**t**	***P***
Knowledge	Gender (compared with male)	female	0.03	0.02	0.07	1.23	0.22
	Hospital grade (compared with Grade II class A)	Grade III class A	0.01	0.01	0.07	1.14	0.26
		Grade III class B	0.02	0.01	0.14	2.50	**0.01**
	Job position (compared with doctor)	nurse	0.02	0.02	0.10	1.55	0.12
	Professional title (compared with primary)	intermediate	0.02	0.01	0.12	2.44	**0.02**
		senior	0.04	0.01	0.14	2.63	**0.01**
	department's emphasis on the prevention and control of MDRO infections (compared with averagely)	relatively	−0.01	0.03	−0.03	−0.16	0.87
		greatly	0.03	0.03	0.18	1.04	0.30
Attitude	Gender (compared with male)	female	0.04	0.03	0.06	1.15	0.25
	department's emphasis on the prevention and control of MDRO infections (compared with averagely)	relatively	−0.08	0.07	−0.20	−1.18	0.24
		greatly	0.01	0.07	0.01	0.07	0.94
	the number of related training per year		−0.001	0.01	−0.01	−0.16	0.87
	willingness to train (compared with no)	yes	−0.003	0.05	−0.003	−0.06	0.96
	carrying out decolonization treatment (compared with no)	yes	0.03	0.02	0.08	1.52	0.13
Practice	Gender (compared with male)	female	0.05	0.01	0.19	3.81	**<0.01**
	Professional title (compared with primary)	intermediate	0.001	0.01	0.01	0.18	0.86
		senior	0.01	0.01	0.03	0.62	0.54
	NICU nosocomial infection control personnel (compared with no)	yes	−0.009	0.01	−0.05	−0.95	0.34
	hospital's emphasis on the prevention and control of MDRO infections (compared with averagely)	relatively	−0.002	0.04	−0.01	−0.04	0.97
		greatly	0.02	0.04	0.13	0.52	0.60
	rules and regulations (compared with no)	yes	0.05	0.03	0.09	1.64	0.10
	hospital's attention to the NICU (compared with no)	yes	0.02	0.04	0.03	0.53	0.60
	isolation ward (compared with no)	yes	<0.001	0.03	0.001	0.02	0.99
	regular supervision by hospital (compared with no)	yes	0.14	0.06	0.12	2.26	**0.03**
	the number of related training per year		0.004	0.002	0.10	2.01	**<0.05 (0.046)**
	willingness to train (compared with no)	yes	0.01	0.02	0.02	0.38	0.71
	carrying out active screening (compared with no)	yes	0.01	0.02	0.03	0.47	0.64
	carrying out decolonization treatment (compared with no)	yes	0.01	0.01	0.03	0.62	0.54

*Knowledge:R^2^=0.10,adjusted R^2^=0.08*.

*Attitude:R^2^=0.06,adjusted R^2^=0.05*.

*Practice:R^2^=0.12,adjusted R^2^=0.09*.

*^†^Knowledge, Attitudes, and Practices*

*^‡^SE – Standard error. The bold values indicate all pvalues p ≤ 0.05 which are statistically significant*.

## Discussion

### The KAP of NICU Doctors and Nurses

This study found that the scoring rate of the knowledge questionnaire was higher than 0.85 (median), and the scoring rate of attitude and practice questionnaire was higher than 0.95 (median), indicating that the KAP of NICU doctors and nurses toward the prevention and control of nosocomial infection with MDRO was generally acceptable, but there was still room for improvement. Based on knowledge, doctors and nurses had a poor grasp of “basic knowledge of MDRO” and “special prevention and control measures.” Hospitals and departments should provide targeted training in these aspects to improve the knowledge of doctors and nurses in the prevention and control of nosocomial infection with MDRO. Our results were similar to those of the study of Kang et al. (23), who found that the mean scoring rate of knowledge of nurses was 0.83, and the scores in basic information and environmental management in terms of knowledge were low.

However, other studies found lower KAP levels of doctors or nurses. The multicenter cross-sectional study of Vaillant et al. (24) conducted in 58 randomly selected French healthcare facilities found that the mean knowledge score was 4.7/8 (SD: 1.3) in medical staff. The study of Zhou et al. (25) found that only 33% and 12% of ICU doctors were familiar with standardized and specialized antimicrobial susceptibility testing (AST) methods, respectively. The study of Lebentrau et al. (26) showed that both urologists and non-urologists had poor knowledge about antibiotic stewardship. The study of Alene et al. (12) revealed that 39.5% of health workers' knowledge score was seven or more, which was considered good, and only 19.6% of the respondents' self-reported practices score were four or more, which was considered good practice. Our study found higher KAP levels of doctors and nurses than these four studies. The possible reasons are as follows: our research used electronic questionnaires instead of paper questionnaires, and it was not possible for participants to fill in and return the questionnaires on the spot, which may lead participants to rate themselves higher in retrospective self-evaluation. In addition, different scales were used in different studies. The sensitivity of different scales to KAP measurement may be different, likely leading to different results.

Our study found that attitude was positively correlated with practice. This was similar to the findings of studies by Wu (21) and Liu et al. (27), which focused on adult wards in Shanxi Province and hospitals of different grades in Neimenggu Province in China, respectively. Therefore, hospitals and departments can improve the prevention and control behavior by holding lectures and other ways to correct the attitude of the medical staff. However, unlike the studies of Wu (21) and Liu et al. (27), our study did not find a significant correlation between knowledge and attitude or between knowledge and practice. The possible reasons are as follows: the attitude of doctors and nurses toward prevention and control is influenced by many other factors besides knowledge, such as their own personality and experience, as well as their professional qualities. The factors that affect the prevention and control behavior of medical staff are more complicated. Even if the knowledge level of medical staff is high but their attitudes are not correct, the implementation of their prevention and control measures is likely to be unsatisfactory.

### Influencing Factors Associated With the KAP

In this study, the influencing factors of KAP were Grade III class B hospital, intermediate and senior title, being female, regular supervision, and training. Our results were similar to those of the study of Liu et al. (27), which found that the KAP level of women was higher than men, and those without professional titles scored the lowest.

Doctors and nurses in senior hospitals had a higher level of knowledge than those in primary hospitals, possibly due to more training and more standardized hospital management. The results of the survey (13) showed that most medical staff in primary medical institutions participated in the training of nosocomial infection control and prevention less frequently, and their mastery of knowledge related to nosocomial infection was worse. The knowledge of medical staff with a intermediate or senior professional title was better, which may be related to longer working time, richer clinical experience, and stronger professional skills. Vaillant et al. (24) also found that one of the factors that correlated with knowledge was professional title (adjusted OR: 3.7, 95%CI: 3.09–4.44).

Women's self-reported behavioral compliance is stated to be higher than men's, possibly because women are more likely to receive continuing education and implement protective measures, while men are more concerned with reducing the cost of infection control (28). Long-term, standardized supervision can act as an external pressure to urge medical personnel to implement prevention and control measures. Therefore, hospitals and departments should strengthen supervision to improve the behavior compliance of prevention and control measures. The current study also found that training can enhance the self-reported behavioral compliance of medical staff, which was similar to the results of several other studies (12, 21, 24). In addition, one study (29) whose interventions included hand hygiene training and certification for medical staff effectively reduced MDRO infection. Training can not only improve the medical staff's grasp of infection prevention and control knowledge, deepen their understanding of MDRO infection prevention and control, and make them realize the importance of prevention and control but also regulate the behavior of doctors and nurses, which is an important means of improving the KAP of medical staff toward MDRO infection prevention and control. Hospitals and departments should organize regular training. The training content should be formulated according to the characteristics of medical staff in the department, and the training methods should include lectures, watching educational videos, and on-site guidance, among others. (18).

## Limitations

There are some limitations to our study. First, this is a cross-sectional study that did not evaluate the changes of the KAP level in the participants over time. Second, the use of self-assessment tools may affect the reliability of the survey, especially when it comes to the practice survey because self-reports cannot tell much about how people actually behave. Finally, the proportion of men and doctors in this study was relatively low, which may make the results of our study unable to truly and comprehensively reflect the actual situation of men and doctors in selected hospitals. In addition, the convenience sampling method does not mark the research subjects as representative of doctors and nurses in Zhejiang Province, so the generality of the research results is limited. Future studies could use random sampling to improve the external validity of the results.

## Conclusions

There is still room for improvement in the KAP of doctors and nurses in NICUs, especially in the knowledge of “basic knowledge of MDRO” and “special prevention and control measures,” The influencing factors of KAP were hospital grade, professional title, gender, regular supervision and training. Men, doctors and nurses in Grade II hospitals, and doctors and nurses with primary professional titles had worse KAP. Training and supervision helped improve the KAP.

## Relevance to Clinical Practice

To improve the KAP of doctors and nurses to enhance the MDRO infection prevention and control effect in NICU, hospitals and departments should carry out targeted training and strengthen supervision, while Grade II hospitals, men, and doctors and nurses with primary professional titles need more attention.

## Data Availability Statement

The original contributions presented in the study are included in the article/supplementary material, further inquiries can be directed to the corresponding author.

## Ethics Statement

The studies involving human participants were reviewed and approved by the Ethics Committee of the Children's Hospital, Zhejiang University School of Medicine (2021-IRB-025). Written informed consent for participation was not required for this study in accordance with the national legislation and the institutional requirements.

## Author Contributions

JZ and SC was responsible for the conception and design of the study, were responsible for the acquisition, analysis, or interpretation of data, completed the final approval of the version to be published, and agreed to be accountable for all aspects of the work in ensuring that questions related to the accuracy or integrity of any part of the work are appropriately investigated and resolved. JZ were responsible for drafting of the manuscript. SC were responsible for critical revision of the manuscript for important intellectual content and directed and supervised the study. All authors contributed to the article and approved the submitted version.

## Conflict of Interest

The authors declare that the research was conducted in the absence of any commercial or financial relationships that could be construed as a potential conflict of interest.

## Publisher's Note

All claims expressed in this article are solely those of the authors and do not necessarily represent those of their affiliated organizations, or those of the publisher, the editors and the reviewers. Any product that may be evaluated in this article, or claim that may be made by its manufacturer, is not guaranteed or endorsed by the publisher.

## References

[1] Giuffrè M Geraci DM Bonura C Saporito L Graziano G Insinga V. The increasing challenge of multidrug-resistant gram-negative bacilli: results of a 5-year active surveillance program in a neonatal intensive care unit. Medicine (Baltimore). (2016) 95:e3016. 10.1097/MD.0000000000003016 26962817 PMC4998898

[2] Silwedel C Vogel U Claus H Glaser K Speer CP Wirbelauer J. Outbreak of multidrug-resistant Escherichia coli sequence type 131 in a neonatal intensive care unit: efficient active surveillance prevented fatal outcome. J Hosp Infect. (2016) 93:181–6. 10.1016/j.jhin.2016.02.014 27117761

[3] Peltier F Choquet M Decroix V Adjidé CC Castelain S Guiheneuf R. Characterization of a multidrug-resistant Klebsiella pneumoniae ST607-K25 clone responsible for a nosocomial outbreak in a neonatal intensive care unit. J Med Microbiol. (2019) 68:67–76. 10.1099/jmm.0.000884 30507374

[4] Jain S Gaind R Kothari C Sehgal R Shamweel A Thukral SS. VEB-1 extended-spectrum β-lactamase-producing multidrug-resistant *Proteus mirabilis* sepsis outbreak in a neonatal intensive care unit in India: clinical and diagnostic implications. JMM Case Rep. (2016) 3:e005056. 10.1099/jmmcr.0.005056 28348778 PMC5330246

[5] Zingg W Hopkins S Gayet-Ageron A Holmes A Sharland M Suetens C. Health-care-associated infections in neonates, children, and adolescents: an analysis of paediatric data from the European Centre for Disease Prevention and Control point-prevalence survey. Lancet Infect Dis. (2017) 17:381–9. 10.1016/S1473-3099(16)30517-5 28089444

[6] Zaidi AK Huskins WC Thaver D Bhutta ZA Abbas Z Goldmann DA. Hospital-acquired neonatal infections in developing countries. Lancet. (2005) 365:1175–88. 10.1016/S0140-6736(05)71881-X 15794973

[7] Seale AC Blencowe H Manu AA Nair H Bahl R Qazi SA. Estimates of possible severe bacterial infection in neonates in sub-Saharan Africa, south Asia, and Latin America for 2012: a systematic review and meta-analysis. Lancet Infect Dis. (2014) 14:731–41. 10.1016/S1473-3099(14)70804-7 24974250 PMC4123782

[8] UNICEF. Committing to child survival: a promise renewed. Progress report 2013. Available online at: http://www.unicef.org/publications/files/APR_Progress_Report_2013_9_Sept_2013.pdf (accessed Dec 4, 2013).

[9] Seale AC Blencowe H Zaidi A Ganatra H Syed S Engmann C. Neonatal severe bacterial infection impairment estimates in South Asia, sub-Saharan Africa, and Latin America for 2010. Pediatr Res. (2013) 74 Suppl 1:73–85. 10.1038/pr.2013.207 24366464 PMC3873707

[10] Xu L Wang RY Chen BB Xie MF Fu ZZ. Risk factors and prevention measures of multiple drug-resistant infections in neonatal intensive care unit. Chin J General Pract. (2018) 16:1314−7.

[11] Caminero JA. Multidrug-resistant tuberculosis: epidemiology, risk factors and case finding. Int J Tuberc Lung Dis. (2010) 14:382–90. 20202293

[12] Alene KA Adane AA Yifiru S Bitew BD Adane A Koye DN. Knowledge and practice of health workers about control and prevention of multidrug-resistant tuberculosis in referral hospitals, Ethiopia: a cross-sectional study. BMJ Open. (2019) 9:e022948. Published 2019 Feb 19. 10.1136/bmjopen-2018-022948 30782870 PMC6368005

[13] Rahiman F Chikte U Hughes GD. Nursing students' knowledge, attitude and practices of infection prevention and control guidelines at a tertiary institution in the Western Cape: A cross sectional study. Nurse Educ Today. (2018) 69:20–5. 10.1016/j.nedt.2018.06.021 30007142

[14] Ogoina D Pondei K Adetunji B Chima G Isichei C Gidado S. Knowledge, attitude and practice of standard precautions of infection control by hospital workers in two tertiary hospitals in Nigeria. J Infect Prev. (2015) 16:16–22. 10.1177/1757177414558957 28989394 PMC5074133

[15] Suliman M Aloush S Aljezawi M AlBashtawy M. Knowledge and practices of isolation precautions among nurses in Jordan. Am J Infect Control. (2018) 46:680–4. 10.1016/j.ajic.2017.09.023 29103636 PMC7132704

[16] Ni P Chen JL Liu N. The sample size estimation in quantitative nursing research. Chin J Nurs. (2010) 45:378–80.CNKI:SUN:ZHHL.0.2010-04-044

[17] The Ministry of Health of China. Technical Guidelines for the Prevention and Control of Nosocomial Infection with MDRO. (2011). Available online at: http://www.nhc.gov.cn/cms-search/xxgk/getManuscriptXxgk.htm?id=50487

[18] Huang X Deng ZD Ni YX Deng M Hu BJ Li LY. Chinese experts' consensus on prevention and control of multidrug resistance organism healthcare-associated infection. Chin J Infect. Cont. (2015) 14:1–9.CNKI:SUN:GRKZ.0.2015-01-001.

[19] World Health Organization. Guidelines on Core Components of Infection Prevention and Control Programmes at the National and Acute Health Care Facility Level. Geneva: World Health Organization (2016). 27977095

[20] World Health Organization. Guidelines for the Prevention and Control of Carbapenem-Resistant Enterobacteriaceae, Acinetobacter baumannii and Pseudomonas aeruginosa in Health Care Facilities. Geneva: World Health Organization (2017). 29630191

[21] Wu T. The Nurse Multi-drug Resistant Organism Infection Prevention Control Knowledge, Attitude, Behavior Investigation Intervention Study[D]. Shanxi Medical University. (2014). Available online at: https://kns.cnki.net/KCMS/detail/detail.aspx?dbname=CMFD201402&filename=1014324531.nh

[22] Shi J Mo X Sun Z. Content validity index in scale development. Zhong Nan Da Xue Bao Yi Xue Ban. (2012) 37:152–5. 22561427 10.3969/j.issn.1672-7347.2012.02.007

[23] Kang J Cho J Kim Y Kim DH Lee J Park HK. Hospital nurses' knowledge and compliance on multidrug-resistant organism infection control guideline. J Korean Acad Nurs. (2009) 39:186–97. 10.4040/jkan.2009.39.2.186 19411790

[24] Vaillant L Birgand G Esposito-Farese M Astagneau P Pulcini C Robert J. Awareness among French healthcare workers of the transmission of multidrug resistant organisms: a large cross-sectional survey. Antimicrob Resist Infect Control. (2019) 8:173. 10.1186/s13756-019-0625-0 31749961 PMC6852912

[25] Zhou JJ Patel SJ Jia H Weisenberg SA Furuya EY Kubin CJ. Clinicians' knowledge, attitudes, and practices regarding infections with multidrug-resistant gram-negative bacilli in intensive care units. Infect Control Hosp Epidemiol. (2013) 34:274–83. 10.1086/669524 23388362 PMC4494664

[26] Lebentrau S Gilfrich C Vetterlein MW Schumacher H Spachmann PJ Brookman-May SD. Impact of the medical specialty on knowledge regarding multidrug-resistant organisms and strategies toward antimicrobial stewardship. Int Urol Nephrol. (2017) 49:1311–8. 10.1007/s11255-017-1603-1 28432607

[27] Liu WP Jiao YY Guo TH Xu BB Jia HJ Hai YT. Nosocomial infection prevention and control knowledge, attitude and practice and influencing factor survey among healthcare workers. Chin J Nosocomiol. (2019) 29:1906–10+1916.CNKI:SUN:ZHYY.0.2019-12-033.

[28] McCarthy GM MacDonald JK. Gender differences in characteristics, infection control practices, knowledge and attitudes related to HIV among Ontario dentists. Community Dent Oral Epidemiol. (1996) 24:412–5. 10.1111/j.1600-0528.1996.tb00890.x 9007360

[29] Qadir M Qamar FN Resham S Ali R Khalil A Ahmed S. Effectiveness of simple strategies in reducing multidrug resistant blood stream infections in Neonatal Intensive Care Unit of tertiary care hospital in Karachi, Pakistan. J Pak Med Assoc. (2015) 65:72−5. 25831680

